# What Is the Best Treatment of the Femoral Shaft Nonunion after Intramedullary Nailing? A Systematic Review

**DOI:** 10.3390/life13071508

**Published:** 2023-07-04

**Authors:** Luca Bianco Prevot, Alessandra Nannini, Laura Mangiavini, Andrea Bobba, Sara Buzzi, Federico Sinigaglia, Giuseppe Peretti

**Affiliations:** IRCCS Galeazzi—S. Ambrogio Institute, EUORR University Equip of Regenerative and Reconstructive Orthopedics, Via Cristina Belgioioso 173, 20157 Milan, Italy; alessandra.nannini@unimi.it (A.N.); laura.mangiavini@unimi.it (L.M.); andrea.bobba@unimi.it (A.B.); sara.buzzi@unimi.it (S.B.); federico.sinigaglia@unimi.it (F.S.); giuseppe.peretti@unimi.it (G.P.)

**Keywords:** pseudarthrosis, nonunion, femoral shaft fractures, bone graft, intramedullary nailing

## Abstract

Nonunion (NU) is one of the most feared complications of femoral shaft fracture treatment. Femoral shaft fracture treatment is often linked with poor bone stock and reduced bone metabolism. In this paper, the goal is to carefully analyze the best treatment options for patients who developed nonunion after the intramedullary nailing of a femoral shaft fracture. A systematic review of the literature available in the PubMed, EMBASE and Cochran library databases was carried out, and 16 studies were included. Exclusion criteria included case reports and case series that do not have data about clinical outcomes or functional outcomes and included fewer than 10 patients. The reviewed data provide evidence for very good results about the treatment of this pathology with exchanging intramedullary nails or the implantation of a plate and screws (general healing rate of 96.3%). Moreover, the data support the utilization of autologous bone graft in order to stimulate the healing process. In conclusion, the choice between these two types of treatment must be guided by the type of pseudarthrosis that the patient presents. Additionally, bone grafting or growth factors promote bone regenerative processes, especially in patients with oligo-atrophic pseudoarthrosis.

## 1. Introduction

Pseudarthrosis in femoral fractures is one of the most difficult-to-treat complications, occurring in less than 1% of operated patients [1]. Nonunion (NU) is a condition characterized by incomplete healing within 9 months of injury or no signs of bone callus formation on subsequent radiographs within 3 months; nonunion can be classified as hypertrophic NUs.

Nonunions can also be divided into septic and aseptic NUs (furthermore they can still be subdivided according to the presence or absence of infection) [2,3]. An increased incidence of pseudarthrosis in femoral shaft fractures has recently been observed due to the increased survival of patients with multiple severe injuries and the widening of indications for intramedullary nailing. The causes most frequently associated with the failure of fracture synthesis and the onset of NUs are both mechanical factors (e.g., insufficient stability of the synthesis) and biological factors (such as severity of soft tissue damage, open fractures, extensive comminution smoking, and diabetes) [4]. Nonunion of the diaphyseal femur can be very difficult to treat and can often have a significant impact on the daily activity and quality of life of patients. The treatment of diaphyseal fractures of the femur is increasingly performed through the implantation of an intramedullary nail. This surgical procedure results in the natural consequence that nonunions after the implantation of the intramedullary nail are more and more frequent. A number of techniques have been proposed for the treatment of femoral shaft nonunion, including electromagnetic fields [5], low-intensity ultrasound [6], shock wave therapy (ESWT) [7], external fixation [8], and internal fixation with plate and screws or with intramedullary nails [9]. The treatment of nonunion with a compression plate and screws, with or without bone grafting, is described in the literature as a valid treatment option, improving the biomechanical conditions at the fracture site without causing significant biological damage that could compromise fracture healing.

Furthermore, after careful analysis, different reports have showed good results after the treatment of femoral nonunions with single- or double-plate osteosynthesis combined with autologous bone grafting [10].

The purpose of this systematic review of the literature is to evaluate the best surgical therapeutic strategies for patients with femoral pseudoarthrosis developing after intramedullary nail fixation surgery; identify the healing rate after surgery; and explore the recovery of functionality of the operated limb during the follow-up period.

## 2. Materials and Methods

This systematic review adheres to the Preferred Reporting Items for Systematic Reviews and Meta-Analyses (PRISMA) guidelines, which provide a systematic checklist for helping reviewers to transparently report the reasons leading to the conducted analysis, its contents and the final findings [11].

The systematic review was registered and allocated in the PROSPERO database (CRD42023424100), National Institute for Health Research, University of York, Center for Reviews and Dissemination.

### 2.1. Inclusion Criteria

During the analysis in the scope of the systematic review, the literature was selected while respecting the criteria outlined below:

Randomized controlled trials (RCTs), non-randomized trials, prospective study, retrospective study, comparative cohort studies, case-control studies, and case series were included. Case reports and case series that did not have data about clinical outcomes or functional outcomes and included fewer than 10 patients were excluded. We also excluded all the studies involving animals or analyzing nonunions involving different bone segments. Studies performed in skeletally mature patients who had undergone surgery for aseptic nonunion of the femoral shaft after intramedullary nail fixation following a femur fracture were considered eligible in the analysis. Studies with a minimum mean follow-up of one year were selected.

This systematic review focuses on nonunions arising at the level of the femoral shaft. This district was considered as the portion of the femur between 5 cm distal to the lesser trochanter and 5 cm proximal to the adductor tubercle.

Outcome measures extracted from the studies were radiological changes, complications, treatment failures, and union rate of the nonunion focus.

Articles dealing with cases of pseudarthrosis in periprosthetic fractures and articles dealing with pseudarthrosis in different sites were excluded.

### 2.2. Database Research

We carried out a systematic search of the significant literature using PubMed, EMBASE and Cochrane Library databases. With the aim of obtaining the data relating to the most recent and updated treatments, we decided to select articles published between 2013 and 2022.

The research was carried out in December 2022.

Our PICO approach (patients/population, intervention, comparator, and outcomes) was defined according to the following question: For patients with nonunions developed after the treatment of femoral shaft fractures with intramedullary nailing (patients/population), which treatment, including intramedullary nail replacement, plate and screw fixation, addition of autologous bone graft, and nail dynamization (operation/comparator) is associated with superior outcomes (results)?

The following search string on the various databases was implemented:

((femur AND fracture*) OR (femoral AND fracture*)) AND (midshaft OR shaft OR diaphyseal) AND (ununited OR union delay OR Fracture Healing OR pseudarthrosis OR delayed union* OR delayed union OR nonunion* OR nonunion* OR nonunion*) AND (management OR treatment).

Two independent reviewers (LBP and FS) assisted in the conduct and validation of the research. Only English written articles were accepted.

### 2.3. Study Selection

Articles that emerged from the research were independently screened by two independent reviewers (LBP and FS). At first, we analyzed the title and, if it was interesting, a more detailed analysis of the abstract was performed. After excluding studies that did not meet the eligibility criteria, we read the whole content of the remaining articles in order to evaluate their eligibility. Disagreement was resolved by group discussion, with senior author arbitration. Studies were not anonymized as to authorship, affiliation, and source. In addition, no attempt was made to contact the authors for individual patient data.

At the end of the process, additional studies that may have been missed were searched for manually by reviewing the reference lists of included studies and related systematic reviews.

### 2.4. Data Collection

Data concerning the patients (age, sex, septic nonunion, follow-up evaluation), type of surgical technique (type of fixation of the first operation, type of fixation of the second operation, use of bone grafts) were extracted for each study. 

The data were extracted from the selected articles using a computerized tool created with Microsoft Access (Version 2010, Microsoft Corp., Redmond, WA, USA).

Due to the heterogeneity of the clinical studies and the population sample analyzed in the various studies, some data were missing or are non-extrapolatable; therefore, they have been considered as missing data in the presentation of our results.

### 2.5. Quality Evaluation

The selected articles were evaluated using the Methodological Index for Nonrandomized Studies (MINORS) score [12]. The checklist includes 12 items, of which the last 4 are specific to comparative studies. Each item was given a score of 0–2 points. After analysis, it was decided to set the optimal score at 16 points for non-comparative studies and 24 for comparative studies.

**Figure 1:**
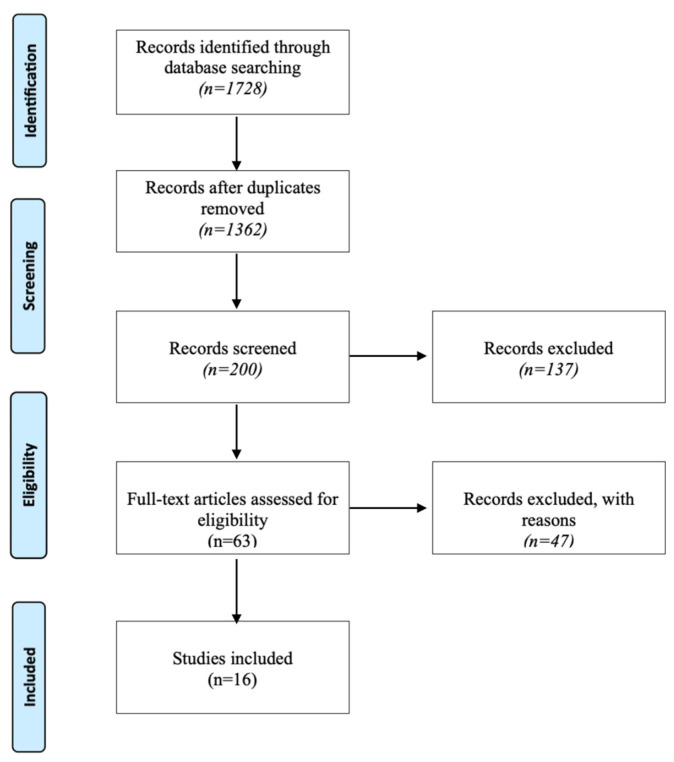
Item selection flowchart.

Figure 1 shows the illustrating flowchart of the selection process of the articles.

## 3. Results

This paragraph is intended to describe the summary statistics underlying the systematic approach followed in this paper. In particular, the informatics research identified 1728 studies. After that, 366 duplicates were deleted, and 1362 studies remained. An additional 1162 studies were discharged after title examination and an additional 137 articles were discarded after reviewing the abstracts, bringing the number to 63 articles. An additional 47 articles were excluded according to inclusion and exclusion criteria. A manual search for articles of was performed, but no additional studies were found.

This resulted in 16 studies for final analysis Figure 1.

### 3.1. Demographics

Of the 16 studies selected, 14 were retrospective [13,14,15,16,17,18,19,20,21,22,23,24,25,26] and 2 were prospective [27,28]. Overall, data from 632 patients were analyzed in our systematic review (summarized data are reported on Table 1).

The average age of the analyzed sample was 37.8 ± 4.4 years; only one study did not report the average age [20].

In all selected articles, patients had a femoral shaft fracture, and all patients underwent internal fixation surgery with intramedullary nail implantation. In particular, a retrograde intramedullary nail was used in 11 cases, while an antegrade intramedullary nail was used in 621 cases.

The most widely used anterograde nail to treat femoral fractures in patients in the selected studies was the Gamma^®^ 3 model, Stryker, Kalamazoo, MI, USA.

In the analyzed studies, a lateral approach to the femur was used for all patients in whom a plate with screws was implanted.

The average time from the fracture event to the nonunion treatment surgery was 20.25 ± 12.48 months.

### 3.2. Surgical Information about NU Treatment

In 162 patients, nonunion was treated with the removal of the intramedullary nail associated with reaming the medullary canal and the reimplantation of a nail of a larger size [20,22,26,27].

Pseudoarthrosis was treated in 18 patients with intramedullary nail revision associated with Poller screw implantation [17]. In 61 patients, nonunion was treated with a revision of the intramedullary nail associated with autologous bone grafting [13,25]. Pseudoarthrosis was treated in 24 patients, with the maintenance of the intramedullary nail associated with plate and screw implantation [20,22], while in 231 patients, the maintenance of the intramedullary nail was associated with plate and screw implantation and autologous bone grafting (in 210 patients) [14,15,16,21,24,26,28] or xenogenic bone grafting (in 21 patients) [18]. In 115 patients, nonunion was treated with an intramedullary nail revision associated with plate and screw implantation and autologous bone grafting [19,22,23]. In total, therefore, 356 patients underwent a revision of the intramedullary nail, while 255 patients did not undergo a revision of the implanted material. Autologous bone graft was utilized in a total of 407 patients (86.6%). In all the articles included in this study, the autologous bone was harvested from the iliac crest ipsilateral to the limb affected by the pseudoarthrosis. 

In the studies included in our review, no platelet derivatives, BMPs, or other biological factors favoring the healing of the nonunion were used.

An article reports the data of patients treated with a xenogenic bone splint [18]. 

### 3.3. Outcomes

A total of 558 of 611 patients (96.3%) achieved bone consolidation, in a mean time of 8.26 ± 6.12. A complete (100%) bone healing was reported in 10 studies [13,16,17,18,19,20,23,24,25,28]; in three studies, bone healing rate was reported between 95% and 100% [15,22,27]; and in three studies, bone healing rate was less than 95% [14,21,26].

The average time in which healing of the nonunion focus occurred was 8.59 ±  6.06 months. In one study [27], 61% of patients had a median healing time of 2–5 months, 21% of patients 5–8 months, and 18% of patients >8 months. In one study, the mean time to healing was not reported [20].

The healing of the fracture was considered by the authors of the articles and analyzed according to clinical signs (absence of pain) and radiographic signs (formation of bone callus). The mean follow-up time was 18.8 ±  7.1 months, while no data regarding the mean follow-up time were reported in three studies [19,21,28]. The analyzed studies report a total of 24 complications, representing approximately 3.8%. In a study of patients treated with intramedullary nail revision with reaming of the intramedullary canal, delayed union was reported as a complication in 14 patients, requiring additional treatment such as nail dynamization, shock wave therapy, and cancellous bone grafting [27]. Other complications were surgical site infection in one patient, surgical wound dehiscence in three patients, pain at the proximal intramedullary nail insertion point in two patients, and implant failure was reported in three patients.

**Table 1:** Summarizes analyzed data.

Studies Included in the Review and Main Features.									
Author and Year	Type of Study	Cases	Age (Mean)	Age (Range)	T1-T2 (Months)	Treatment	Union Rate (%)	Time of Union (Months)	Follow-up (Months)
**Wu et al., 2022 [13]**	Re	48	38	19-67	50	EX NAIL + ABG	100	3.4	32
**Hierholzer et al., 2014 [27]**	Pr	72	46	18–69	11 in 46% of cases >12 in 34% of cases <6 in 20% of cases	EX NAIL	98	2–5 in 61% of cases 5–8 in 21% of cases >8 in 18% of cases	14
**Saliba Uliana et al., 2019 [14]**	Re	22	32	NS	11.07	AUG PLATE + ABG	86	11.7	23.5
**Park et al., 2013 [15]**	Re	39	41.9	17–68	19.03	AUG PLATE + ABG	97	6.1	24.8
**Lu et al., 2022 [16]**	Re	22	40.8	19–61	NS	AUG PLATE + ABG	100	5.7	18.8
**Kim et al., 2017 [17]**	Re	18	46.8	15–78	7.08	EX NAIL + Poller screw	100	7.5	17.1
**Dai et al., 2015 [18]**	Re	21	34.8	18–62	NS	AUG PLATE + XBG	100	6.2	13.2
**Wang et al., 2014 [19]**	Re	21	40	21– 61	NS	EX NAIL + AUG PLATE + ABG	100	6	NS
**Ru et al., 2013 [20]**	Re	28	NS	NS	28	11 cases EX NAIL 17 cases AUG PLATE	100	NS	18.6
**Gao et al., 2013 [21]**	Re	47	37	18–74	47	AUG PLATE + ABG	92	6	NS
**El Zahlawy et al., 2019 [28]**	Re	34	36.6	17–56	34	AUG PLATE + ABG	100	6.3	NS
**Jhunjhunwala et al., 2015 [22]**	Re	40	35	18–65	6	9 cases EX NAIL 7 cases AUG PLATE 24 cases EX NAIL + AUG PLATE + ABG	97.5	4	12
**Sancheti et al., 2017 [23]**	Re	70	40.7	18–81	18.07	EX NAIL + AUG PLATE + ABG	100	16.7	31.37
**Mohamed et al., 2022 [24]**	Re	20	32.4	18–55	12	AUG PLATE + ABG	100	4.9	13
**Alam et al., 2019 [25]**	Re	13	39.08		13	EX NAIL + ABG	100	26.9	12
**Lai et al., 2019 [26]**	Re	96	31.77 AUG PLATE + ABG 35.79 EX NAIL	NS	NS	26 cases AUG PLATE + ABG 70 cases EX NAIL	70.8	7.57 AUG PLATE + ABG 10.02 EX NAIL	11.89 AUG PLATE + ABG 13.7 EX NAIL

Main features of the studies included in the review. ABG: autologous bone graft, EX NAIL: exchange nail, NS: not specified, PLATE: augmentative plate, Pr: prospective, Re: retrospective, T1-T2: time passed between first treatment and the diagnosis of nonunion, XBG: xenobiotic bone graft.

## 4. Discussion

The purpose of this systematic review was to identify the possible treatments and their success rates in nonunions of femoral shaft fractures initially treated with an intramedullary nail.

The annual incidence of femoral shaft fractures is approximately 10 per 100,000 people [29], and the gold standard of their treatment to date is intramedullary nail fixation [30]. The incidence of PSA in these patients is between 1.9 and 5% [1], and the treatments that can be offered to these patients range from exchange nailing, to dynamization of the nail, to synthesis with plate and screws, to external. All these methods may or may not be associated with autologous or bank bone grafting or growth factors to promote the healing of the nonunion focus [31].

Of fundamental importance is the evaluation of the type of pseudarthrosis: in the case of hypertrophic pseudarthrosis, the pathogenesis is to be found in the poor stability of the fracture site; therefore, treatments that confer greater stability to the fracture will be preferred (e.g., plate and screws or replacement of the nail with a larger one), while if an oligotrophic or atrophic pseudoarthrosis is found, the pathogenesis will be linked to a probable reduction in the biological stimuli for the healing, and therefore it will be appropriate to intervene with procedures that promote the reactivation of the normal reparative processes (e.g., revision of the intramedullary nail with reaming of the intramedullary canal; plate and screws associated with bone graft implantation) [32].

From the selected studies, it can be seen that the most-used type of intervention for the treatment of the pseudarthrosis of diaphyseal fractures of the femur initially treated with an intramedullary nail is the implantation of plate and screws. This intervention is particularly advantageous as it allows us to increase the stability at the level of the fracture site, but at the same time, through the implantation of autologous tissue taken from the patient, to provide an adequate biological stimulus for the fracture healing processes to take place. After analyzing the data that emerged from our review, we found that excellent results are also obtained with the exchanging nail method, i.e., the removal of the previously implanted nail, the reaming of the medullary canal, and the reimplantation of an intramedullary nail with a larger diameter.

From the selected studies, it can be seen that, generally, the nonunions of the femur are not operated on quickly (average of 20.5 months between the fracture and the operation for the nonunion).

The data analyzed show how the treatments for pseudoarthrosis are effective, with the percentage of patients healed being 96.3% and the percentage of complications being around 3.8%.

### 4.1. Nail Revision 

One of the treatment options for nonunions that develop in patients with femoral shaft fractures initially treated with intramedullary nailing is nail removal, and, after reaming the medullary canal, the implantation of a larger diameter intramedullary nail. The effects of nail revision are both biological and mechanical [33].

The biological effects consist in the fact that reaming the medullary canal increases periosteal blood flow while decreasing endosteal vascularization. The increase in periosteal blood flow promotes a callus formation reaction. In addition, the reaming of the intramedullary channel promotes the formation of material containing osteoblasts, stem cells, and growth factors, which play fundamental roles in bone healing. The mechanical effects would be attributed to the fact that the larger diameter of the nail (preferably 2 mm thicker) provides a greater stiffness to the fixture and strength than the original nail. Greater stability can also be achieved by using a longer nail [34]. In hypertrophic nonunions treated with nail replacement, the increased stability will be sufficient for healing. For atrophic nonunions, reamed debris is thought to enhance bone healing. In the analyzed literature, the studies that treated patients with intramedullary nail replacement reported a low rate of complications and cure levels—for example, 98% in the article by C Hierholzer [27], 100% in the article by I. Ru [20], and 97.5% in the article by H. R. Jhunjhunwala. The failure of the nail revision has been observed in cases of pseudarthrosis with large comminution, large segmental defects, and meta-diaphyseal pseudarthrosis [17,20]. In the literature, the rates of persistent nonunion after this procedure are between 11.1% and 46% [35,36]. Another treatment that is proposed in the articles examined for this review is the maintenance of the intramedullary nail associated with the implantation of plates and screws. The rationale of this treatment lies in the ability of the nail to distribute the compression forces at the level of the nonunion focus, and the plate and the screws have the task of further stabilizing the focus and protecting it from transversal and rotational forces.

### 4.2. Dynamization of the Nail

The dynamization of the intramedullary nail represents a valid treatment option for the nonunion of femoral shaft fractures. The dynamization of the intramedullary nail causes an increase in compression at the fracture site by increasing the contact area of the abutments, improves osteogenic processes, and promotes an increase in the transmission of compressive forces at the level of the fracture site, which is essential for the stimulation of osteogenic processes [37]. Nail dynamization surgery is characterized by having low invasiveness, low morbidity, and low cost, making it a viable option compared to bone grafting, exchange nailing, and compression plating. Dynamization, however, is not routinely needed for fracture healing and is associated with a risk of shortening, particularly in oblique or high comminution fractures, and a loss of fracture reduction. The examined literature highlights a rate of nonunion healing after nail dynamization that is approximately 66.4% (24% to 99%) [38].

### 4.3. Plates and Screws

The plate and screw fixation system is a fundamental component in the treatment of femoral shaft pseudoarthrosis; it can be used both as a neosynthesis after the removal of the intramedullary nail (plate fixation) and as an additional means of fixation, leaving the intramedullary nail in place or replacing it after reaming the medullary canal (augmentative plate fixation). This procedure may or may not be associated with the use of bone grafts or local growth factors. The augmentation plate fixation technique improves the biomechanical conditions in the fracture site by removing the rotational instability that can occur following fixation with an intramedullary nail [39]. The results obtained from the studies we analyzed, in which plate and screw implantation was associated with the maintenance or replacement of the intramedullary nail, showed a high rate of bone consolidation (over 96.8%) and an average healing time of 7.3 months. This healing time is short in consideration of the fact that nonunion is a complication that takes many months to achieve complete bone consolidation.

This approach utilizes the load sharing capability of the nail with axial and flexural strength, while the plate resists lateral and rotational forces; moreover, the surgical approach provides a good exposure of the pseudoarthritic focus, with the possibility of performing the cruentation of the fracture stumps with the removal of the fibrous or non-vital tissue and of performing sequestrectomies [21]. However, it is also essential to underline how the intervention of plate and screw implantation very often requires the execution of large skin incisions, extensive muscle dissections, and the detachment of large portions of the periosteum. Deperiosteal surgery is a maneuver that can cause vascular damage to the underlying bone, resulting in inadequate biological stimulation for fracture healing. Therefore, the implantation of the plate and screws must spare the tissues around the fracture as much as possible in order to guarantee an adequate vital environment that promotes the formation of bone callus. Another important advantage of plate and screw implantation is the possibility of performing bone grafts [39,40]; in fact, in 86.6% of cases, the authors of the articles selected in our systematic review report the use of autologous bone harvested from the iliac crest. Prior to the availability of angular stable plates (which can rely on unicortical fixation), this technique was quite challenging due to the need to use bicortical screws. However, angular stable plates have substantially facilitated augmentation plate fixation from a surgical technique point of view [41]. This technique has some disadvantages, in particular the fact that it is a very invasive surgical technique involving a large incision and an extensive approach, with significant compromise of the soft and vascular tissues.

In a meta-analysis by Hua Luo [42], in which the results between the change of the nail and the implantation of the plate and screws were compared, it emerged that the implantation of the plate and screws is superior to the replacement of the nail. In particular, the implantation of the plate and screws has a lower nonunion rate, shorter union time, less intraoperative blood loss, and shorter operative time.

### 4.4. Bone Grafting and Biology Adjuvants 

The literature examination in scope of this review highlights the importance of the use of bone grafting or a biological adjuvant substance associated with surgery in order to augment the healing rate of patients with femur shaft nonunion. Bone grafts have several properties: Osteoconduction refers to the ability of the implanted scaffold to stimulate the internal growth of blood vessels and mesenchymal cells. Among the osteoconductive scaffolds most frequently used in the treatment of nonunions are cements made of calcium sulfate and calcium phosphate. Osteoinduction is the process by which the mesenchymal cells are stimulated to undergo a process of differentiation into the different bone cell series (chondroblasis, osteoblasts, osteocytes); this is a fundamental process for the bone graft, which promotes the formation of new bone through the ossification process [43]. Osteogenesis, on the other hand, is the ability to form new bone by cells derived from the graft. This property is unique to autografts or allografts only. Autologous bone grafting consists of taking bone material from one anatomical site and then transplanting it to another site in the same patient. This type of graft has high osteoinductive, osteoconductive, and osteogenic potential. Among the various autologous grafts, the spongy one is the most commonly used. Its high concentrations of osteoblasts and osteocytes give it high osteogenic potential, and its large trabecular surface induces vessel invasion and incorporation into the receiving site [44]. The literature examination underlines how the best biological adjuvant in patients with femoral nonunion is a cancellous autograft, although its use is still controversial and it is strongly linked with the type of nonunion to be treated. Generally, autologous bone grafting is not necessary in hypertrophic nonunions, as the main cause of these complications an initial fixation of the fracture, which does not guarantee adequate stability. However, many surgeons tend to also use bone grafting in hypertrophic nonunion [32]. This acts as a void filler in cases of pseudarthrosis developing on fractures with high comminution and bone loss, but at the same time, bone grafting also provides all the required biological stimuli, including a high source of viable autologous osteogenic cells residing in bone and bone marrow. Often, most surgeons tend to curettage the nonunion site by removing fibrotic or necrotic tissue, which can leave bone defects of various sizes that require grafting. Although an iliac crest graft is typically used to fill a defect, alternative techniques are available for obtaining tissue for graft [45]. Another device that can be used is the reamer-irrigator-aspirator, which allows bone to be taken as a ream from inside the femoral canal. This technique allows us to obtain bone material that acts both as a filler of voids and as a biological stimulus, being a source of autologous vital osteogenic cells [46].

Given the available data, it is not possible at the moment to state with certainty which is the best treatment for patients suffering from nonunions in femur shaft fractures.

However, both the implantation of plates and screws and the change of the intramedullary nail show excellent results. The topic of the best treatment of nonunions of shaft fractures of the femur requires numerous and further prospective and randomized controlled studies, in particular to evaluate the impact that the new biological therapies (stroma vascular fraction, BMP, pallet rich plasma) can have in promoting the healing of this pathology, especially for oligo-atrophic nonunions in which there is a lack of biological stimulation for healing.

This review certainly has some limitations, including the non-specification in many studies of whether it was atrophic, hypotrophic, or hypertrophic pseudoarthrosis, which is essential information for evaluating the best treatment option; the heterogeneity of the studies included in the review; the retrospective nature of most of them; the lack of control groups; the low volume of data; and the very small sample size in some studies.

## 5. Conclusions

Pseudoarthrosis after the treatment of diaphyseal fractures of the femur represents one of the most fearsome complications and is among the most difficult to manage. The therapeutic choices that can be used are different. Both plate and screw augmentation and the revision of the intramedullary nail with reaming of the medullary canal are valid treatments with excellent chances of healing the nonunion. The choice between these two types of treatment must be guided by the type of pseudarthrosis that the patient presents: the nonunion after nailing is in most cases caused by instability (hypertrophic pseudarthrosis), which is treated by providing stability (for example, by plate implantation as an augment), while in the case of oligotrophic or atrophic pseudarthrosis, the revision of the nail with the reaming of the intramedullary canal represents a valid therapeutic option as it stimulates the biological recovery of the normal healing processes of fractures. Even the dynamization of the nail, especially in hypertrophic pseudoarthrosis, can be a treatment option to consider by virtue of the low invasiveness and the good results it presents. A fundamental factor to be stressed is the use of autologous bone graft or growth factors to promote bone regenerative processes, especially in patients with oligo-atrophic nonunion, which can be used both in the case of an augmentation procedure with plate and screws and in the case of nail revision. Autologous bone grafting could also be considered in those patients who, despite having a hypertrophic nonunion, present risk factors or comorbidities that can slow down the healing processes of the nonunion, e.g., diabetes and cigarette smoking. This only allows for a descriptive statistical analysis without the ability to draw definitive conclusions.
